# CD44 Sorted Cells Have an Augmented Potential for Proliferation, Epithelial-Mesenchymal Transition, Stemness, and a Predominantly Inflammatory Cytokine and Angiogenic Secretome

**DOI:** 10.3390/cimb43010034

**Published:** 2021-06-21

**Authors:** Shankargouda Patil

**Affiliations:** Department of Maxillofacial Surgery and Diagnostic Sciences, Division of Oral Pathology, College of Dentistry, Jazan University, Jazan 45142, Saudi Arabia; dr.ravipatil@gmail.com

**Keywords:** cancer stem cells, CD44, oral squamous cell carcinoma, secretome

## Abstract

Cancer stem cells (CSCs) have garnered attention with their potential for early diagnosis and prognosis of oral squamous cell carcinoma (OSCC). It is still indistinct whether CSCs are recognized with a specific set of characteristics. The present study aimed to assess the association of CD44 with stemness-related, Epithelial Mesenchymal Transition EMT-related genes and the secretome of the CSCs. The single-cell suspension from primary OSCC tumors was prepared by enzymatic digestion and the cells were cultured in-vitro. The cancer stem cells were isolated by CD44+ selection using magnetic cell-sorting. The expression of CD44, proliferation rate, gene expression of EMT-related transcription factors, stemness markers, cytokine levels and angiogenic factors in both cell population was assessed. The sorted CD44+ cells showed significantly higher proliferation rate than heterogenous population. The CD44 expression was >90% in the sorted cells which was higher than the heterogenous cells. The CD44+ CSCs cells demonstrated significant increased levels of EMT-related genes TWIST1 and CDH2 (*N*-cadherin), CSC-related genes CD44 and CD133 (PROM1), stemness-related genes OCT4, SOX2, inflammatory cytokines IL-1ß, IL-12, IL-18 and TNF-α and angiogenic factors Angiopoietin-1, Angiopoietin-2, bFGF and VEGF while levels of epithelial gene CDH1 (E-cadherin) decreased in comparison to mixed cell population. The genetic and secretome profiling of the CD44+ CSCs could serve as diagnostic and prognostic tools in the treatment of oral cancers.

## 1. Introduction

Head and neck cancers (HNCs) are predominant in Asia and oral squamous cell carcinoma (OSCC) in particular accounts for nearly 90 percent of cancer-related fatalities [1]. Early diagnosis of cancer-related symptoms is encouraged to understand and develop strategies for treatment of OSCCs. The prime time for medical intervention is the point where cancer stem cell (CSC)-like cells start appearing. CSCs not only provide tumor heterogeneity but also play a vital role in the progression and metastasis of tumor, drug and radiation resistance, and relapse [2]. As tissue-resident stem cells, CSCs are also responsible for continuous self-renewal and self-propagation over a long period of time [3,4]; hence, detailed analysis of stemness and CSCs is essential in oral precancerous lesions.

The severity of dysplastic changes in premalignant lesions are concomitant with inferior clinical consequences, but more demanding approaches are required to envisage the tumor forming prospects of these premalignant lesions with respect to CSCs and stemness markers. Tumors frequently encompass ordered provisions of gene-wide or epigenetic divergent cancerous and non-cancerous cellular processes [5,6]. In these instances, some speculative phenotypes of tumor-forming cells are being identified through exhaustive experimental work with single-cell suspensions from tumors, xenotransplantation studies and phenotypic cell-surface antigens [7,8]. The attainment of a genetic makeover to tumor-forming ability by CSCs is a collective route that would be apparent in premalignant tissues. The relationship between proclaimed stem cell markers with the oral epithelial tumorigenicity should be studied thoroughly.

Most tumors, including OSCCs, are populated with specialized oncogenic cells positive for CSC markers such as CD44, PROM1, ABCG2, and ALDH-1 [9,10] and demonstrate a superior capability to develop a tumor when transplanted into other animals [11,12]. Recently, CD44, a surface glycoprotein, was highly implicated in the regulation of tumor progression, invasion, and metastasis [13,14]. However, few studies have reported CD44 expression in oral tumors. The correlation between the various grades of OSCC tumors and the regulation by CD44 might reflect cellular invasion and progression in early malignant transformation of the oral region. Further, this correlation can also likely be directly proportional to the degree of cellular differentiation associated with motility and incursion of the lesion [15]. Thus, CD44 could be effective in determining the severity of malignancy in OSCC.

In this study, we have attempted to investigate the expression of potential CSC-marker CD44, pluripotency-associated markers such as OCT4, SOX2, CD133 (PROM1), and EMT-related markers TWIST, ECAD, NCAD and their relationship with secretion of angiogenic factors, inflammatory cytokines, and proliferation of cells in OSCC tumors.

## 2. Materials and Methods

### 2.1. Sample Collection and Ethical Permissions

Primary tumor samples from histopathologically confirmed well-differentiated OSCC patients having history of tobacco chewing and cigarette smoking were obtained (*n* = 5) (Age: 44–58 years; Gender: all males) with appropriate well-versed patient consent. Tumor samples were collected in a sterile container containing complete culture media (DMEM + FBS) (Invitrogen, Carlsbad, CA, USA) and immediately processed. Scientific Research (IRB)- College of Dentistry, Jazan University (reference no-19239) approved the study on 14 April 2020.

### 2.2. Preparation of the Single-Cell Suspension

Tumor samples were carefully washed with phosphate-buffered saline (PBS) (Sigma, St. Louis, MO, USA) containing antibiotics-antimycotic solution (Sigma-Aldrich, St. Louis, MO, USA). Tissue samples were minced using sterilized surgical scissors and subjected to enzymatic digestion with a mixture of enzymes containing 0.4% collagenase I (MP Biomedicals LLC, Santa Ana, CA, USA) and 0.2% dispase II (Roche Diagnostics GmbH, Mannheim, Germany). After incubating at 37 °C for 30 min, the enzyme action was halted by adding fetal bovine serum (FBS) (Gibco, Rockville, MD, USA); following this, the mixture was passed through a 70-micron cell-strainer (Corning, NY, USA). The cells obtained from the mixture were plated in the tissue culture flask for each individual sample separately and fed with cell culture medium (DMEM + FBS).

### 2.3. Magnetic Sorting of CD44+ Cells and Flow Cytometry Analysis for CD44 Marker

Total tumor cells for each individual sample were subjected to magnetic cell sorting independently using CD44 (anti-human) MicroBeads (Miltenyi Biotec, Bergisch Gladbach, Germany) per the protocol provided by the manufacturer. The sorted CD44+ cells were stained with Anti-CD44-PE antibody (Miltenyi Biotec, Auburn, CA, USA) and acquired on a flow cytometer (Attune NxT, Thermo Fisher Scientific, Waltham, MA, USA) to check the purity and seeded with complete media (DMEM + 10% FBS) for further experimentation.

### 2.4. Carboxyfluorescein Succinimidyl Ester (CFSE) Cell Proliferation Assay

Cell proliferation was assessed by flowcytometry using the CellTrace CFSE Cell Proliferation Kit (Invitrogen, Waltham, MA, USA). The experiment was performed at 48 h and 72 h of incubation with complete growth medium. Briefly, 1 µL of CellTrace stock solution in DMSO was added to the cell suspension in PBS for a final working solution. Cells were incubated for 20 min at room temperature or 37 °C, protected from light. The reaction was stopped by adding a large volume of culture medium (6 times the initial volume) to the cells and incubated for 5 min. The cells were pelleted by centrifugation and resuspended in the fresh pre-warmed complete culture medium. This was followed by incubation for at least 10 min before analysis to allow the CellTrace reagent to undergo acetate hydrolysis, and subsequently seeded into the culture dishes with different media compositions. After incubation for 48 h and 72 h, the cells were acquired on the flow cytometer.

### 2.5. RT-qPCR for Stemness and EMT Related Transcription Factors

Total RNA was isolated from cells of both the experimental groups using the GeneJET RNA Purification Kit (Thermo Scientific, Vilnius, Lithuania) following manufacturer’s protocol. The experiment was performed at 48 h of incubation with complete growth medium. For culture expanded cells, passage 4 cells were used. Synthesis of cDNA was performed with the cDNA Reverse Transcription Kit (High Capacity, Applied Biosystems, Carlsbad, CA, USA) and diluted for RT-qPCR as suggested in the manufacturer’s instructions. The amplification of cDNA was carried out with SYBR Green Master Mix (Applied Biosystems, Austin, TX, USA) for OCT4, SOX2, CD44, CD133 (PROM1), TWIST1, CDH1, and CDH2 (IDT, Coralville, IA, USA) on a QuantStudio 5 real-time PCR system (Applied Biosystems, Foster City, CA, USA). The PCR program is given in Table 1. ACTIN served as housekeeping gene. The cycle threshold (*C*_t_) values for each gene were corrected by using the mean *C*_t_ value. mRNA levels were calculated by the ΔΔ*C*_t_ method and were quantified by using the 2^–ΔΔ*C*t^ method, normalized to the average CT for the ACTIN gene expression levels. Primers for the genes are given in Table 2.

### 2.6. ELISA for the Quantitative Analysis of Cytokines and Growth Factors

The analysis of cytokines IL-12, IL-18, IL-1β, and TNF-α and angiogenic factors Angiopoietin-1, Angiopoietin-1, FGF-basic, and VEGF at the protein level was carried out by using KRIBIOLISA human ELISA kits (Krishgen Biosystems, Los Angeles, CA, USA). The experiment was performed at 48 h of incubation with complete growth medium. The conditioned media from cells was obtained after 48 h of incubation in complete medium and the protocol was performed conferring to the experimental instructions provided with the kit. The absorbance was read at 450 nm on a spectrophotometer (Multiskan FC, Thermo Scientific, San Jose, CA, USA).

### 2.7. Statistical Analysis

The data obtained were designated as the means ± standard deviations of the three independent experimental values individually for all 5 samples. Statistics of the data was performed by paired two-samples t-test on GraphPad Prism software (version 8), GraphPad Software, La Jolla, CA, USA; *p* < 0.05 was measured as statistically significant (*p < 0.05 and ** *p* < 0.01).

## 3. Results

### 3.1. CD44 Expression and Proliferation Rate of Sorted CSCs Versus Heterogeneous Population

**Figure 1:**
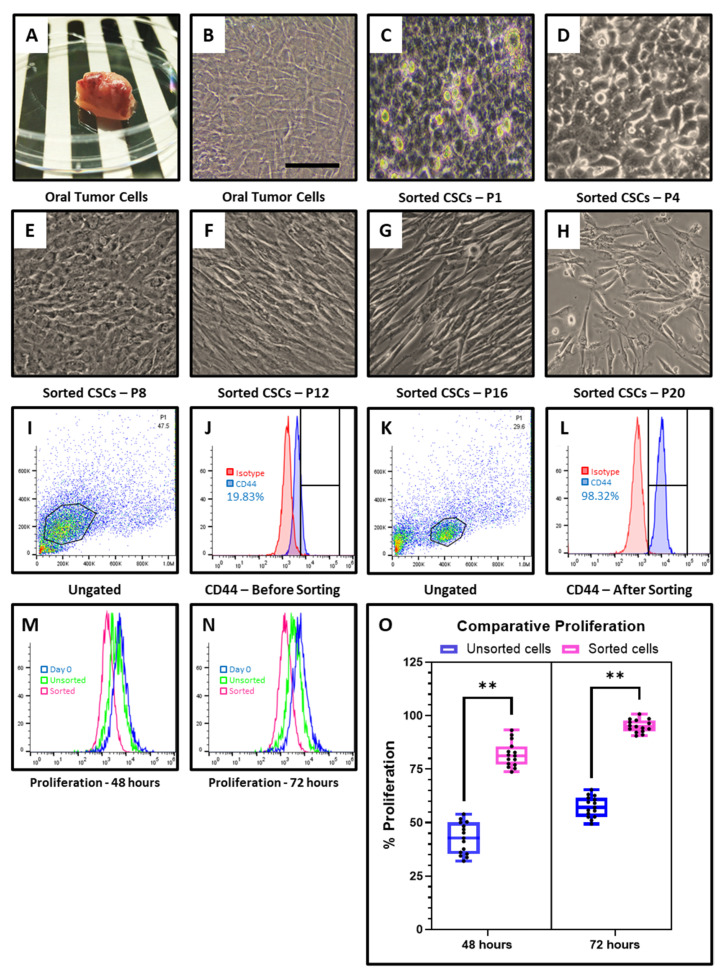
Preparation of the single cell suspension from OSCC tumor was carried out and the cells were subjected to flow cytometry and in vitro expansion. (**A**) OSCC tumor tissue. (**B**–**H**) In vitro cultured primary OSCC cells and sorted CD44+ cells at passage 1 to passage 20. Scale bar = 100 µm. (**I**–**L**) Flow cytometry analysis of OSCC cells and sorted CD44+ cells for expression of CD44 marker. Red: Isotype control; Blue: CD44 positive. (**M**–**O**) Comparative cell proliferation by CFSE assay. Blue: Day 0 MFI; Green: Unsorted MFI; Red: Sorted MFI. Experiments were performed in triplicates for all five samples independently. ns not significant, ** *p* < 0.01.

CD44 expression of the CD44+ sorted CSCs was >90%, whereas the heterogeneous population exhibited ~20% positivity (Figure 1I–L). Moreover, the morphological features of the CD44+ sorted CSCs were more homogenous and had a different appearance in comparison with the total tumor cells (Figure 1A–C). Moreover, the CD44+ sorted CSCs were continuously passaged and observed up to passage 20. These cells showed some significant morphological changes upon long term culture (Figure 1D–H). Late passage cells after passage 8 were looking elongated shaped. Considering the mitotic rate, CD44+ cells exhibited significantly higher proliferation rate at 48 and 72 h when cultured in vitro in comparison with the heterogeneous population (Figure 1M–O).

### 3.2. Gene Expression of CD44+ OSCC Cells and Heterogeneous Tumor Cell Population

**Figure 2:**
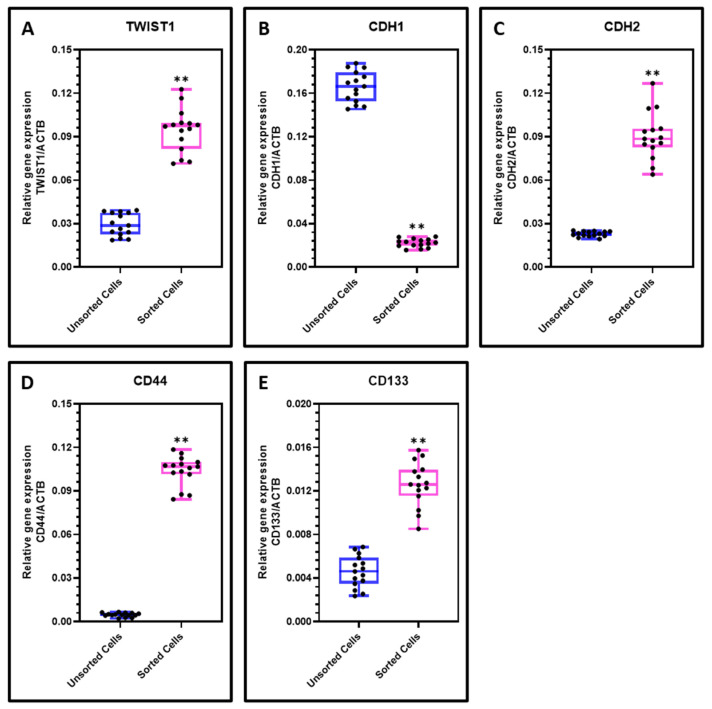
RT-qPCR analysis for gene expression. (**A**–**C**) Comparative analysis of EMT related genes TWIST1, CDH1, and CDH2 in OSCC cells and sorted CD44+ cells; (**D**,**E**) Comparative analysis of CSC markers CD44 and CD133 (PROM1) in OSCC cells and sorted CD44+ cells. Experiments were performed in triplicates for all five samples independently. ns not significant, ** *p* < 0.01.

CD44+ CSCs cells demonstrated a significant increased levels of EMT-related genes TWIST1 and CDH2 (*N*-cadherin) and decreased levels of epithelial gene CDH1 (E-cadherin) in comparison with the heterogeneous OSCC cell population (Figure 2A–C). Considering the expression of CSC-related genes CD44 and CD133 (PROM1) the CD44+ sorted cells demonstrated increased expression than the mixed population of OSCC tumor cells (Figure 2D,E).

### 3.3. Expression of Stemness Genes during In Vitro Expansion of CD44+ Oral Tumor CSCs

**Figure 3:**
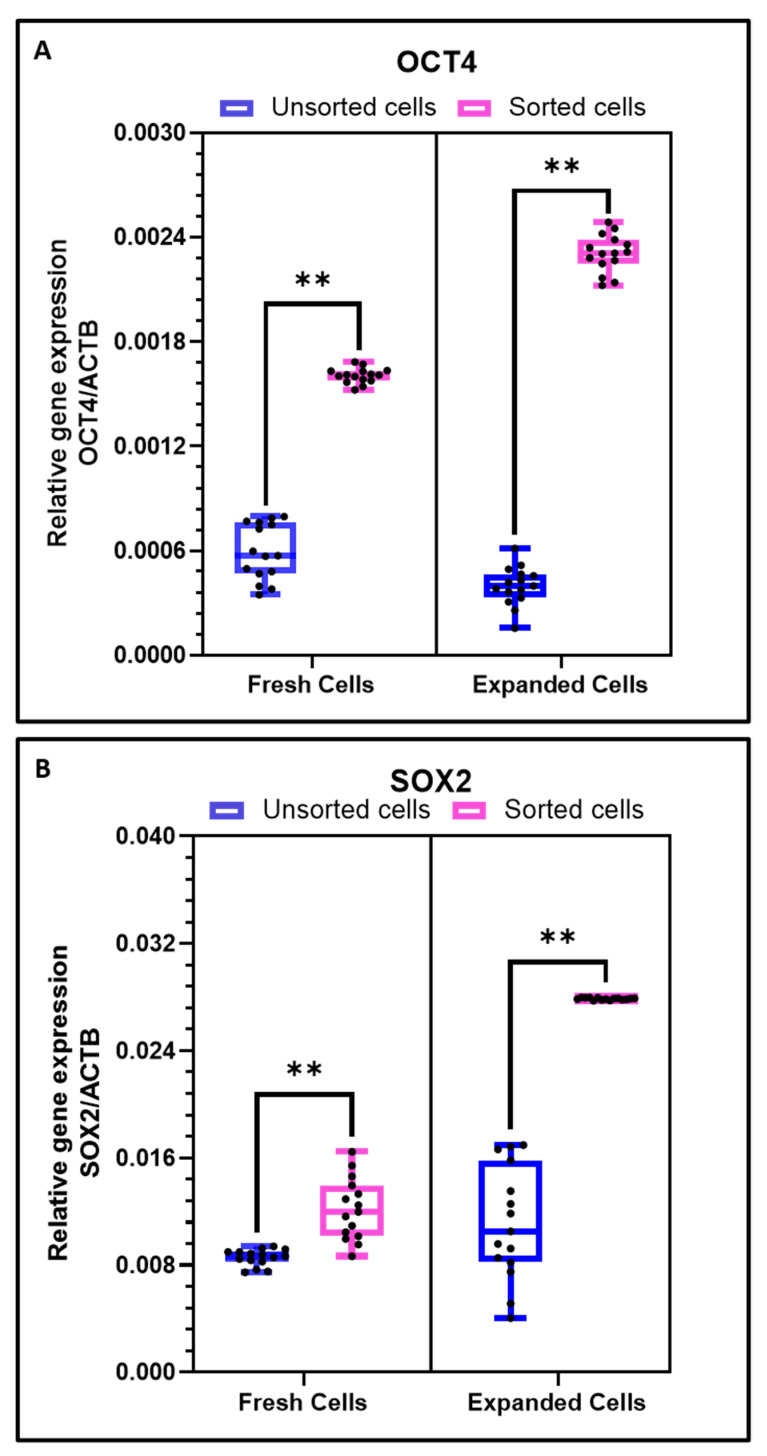
Comparative gene expression analysis of freshly isolated cells and culture expanded cells. (**A**) Comparative gene expression analysis of stemness gene OCT4 in OSCC cells and sorted CD44+ cells; (**B**) Comparative gene expression analysis of stemness gene SOX2 in OSCC cells and sorted CD44+ cells. Experiments were performed in triplicates for all five samples independently. ns not significant, ** *p* < 0.01**.**

Upon in-vitro cultivation and several passaging of the CD44+ sorted OSCC cells, a time-dependent significant increase in the expression of stemness-related genes (OCT4 and SOX2) was observed at passage 4. However, considering the heterogeneous tumor population, the increase in the expression of these genes was not notable. (Figure 3A,B).

### 3.4. Levels of Inflammatory Cytokines and Angiogenic Factors in Sorted CD44+ CSCs and Heterogeneous Tumor Cell Population

**Figure 4:**
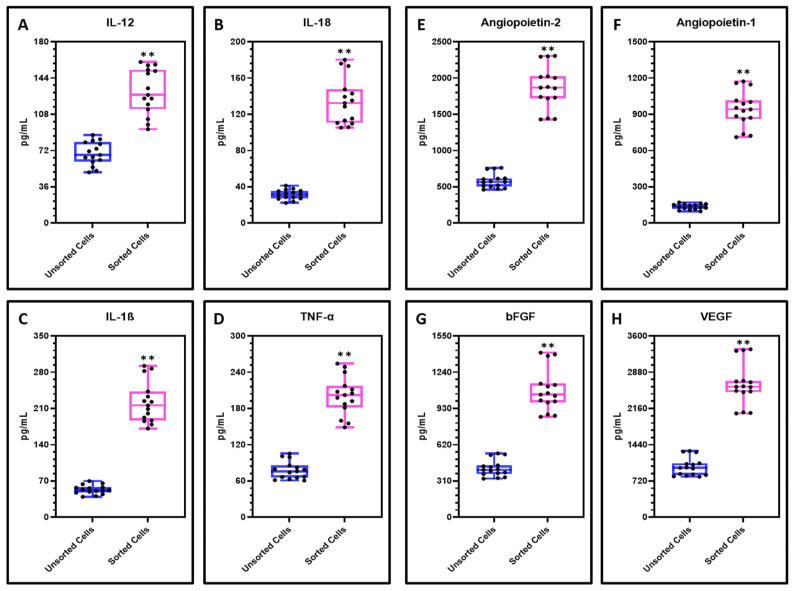
Analysis of cytokines and angiogenic factors in the secretome of OSCC cells by ELISA. (**A**–**D**) Comparative cytokine analysis of inflammatory cytokines IL-12, IL-18, IL-1ß, and TNF-α in the secretomes of OSCC cells and sorted CD44+ cells; (**E**–**H**) Comparative growth factor analysis of angiogenic factors Angiopoietin-2, bFGF, Angiopoietin-1, and VEGF in the secretomes of OSCC cells and sorted CD44+ cells. Experiments were performed in triplicates for all five samples independently. ns not significant, ** *p* < 0.01**.**

The CD44+ CSCs were found to secrete a significantly higher levels of inflammatory cytokines IL-1ß, IL-12, IL-18 and TNF-α compared with the heterogeneous cell population (Figure 4A–D). Considering the angiogenic factors Angiopoietin-1, Angiopoietin-2, bFGF and VEGF, sorted CD44+ cells were found to secrete significantly higher levels than the unsorted cells (Figure 4E–H).

## 4. Discussion

Despite OSCC being associated with high morbidity and mortality, early diagnosis and appropriate treatment may lead to better prognosis. In this regard, contemporary advancement in sophisticated technologies such as salivary proteomics, miRNA analysis, single nucleotide polymorphism, and genome-wide association studies would promote identification and development of distinct markers to predict and treat early-stage OSCCs [4,15,16]. With regular histopathologic examinations, these validated biomarkers would bring about a better diagnosis and prospects for successful therapies when the tumor progression can be intervened with. This would benefit further investigations into molecular pathways of OSCCs used to identify novel therapeutic targets.

A plethora of molecular mechanisms have been established linking CSC population and tumor development and aggression in HNCs. Most recent studies have revealed that the development of a tumor is sustained by a robust population of CSCs in vivo, while differentiating tumor cells contribute very briefly to tumor development. Nevertheless, it is still indeterminate if all tumor types hold such a tiered association, and if during tumor development, all existing cancer cells exhibit long-lasting self-renewing capability like CSCs [17,18].

In our investigations, a comparative gene expression analysis revealed that the sorted CD44+ OSCC CSCs possess higher gene expression of CSC-related genes CD44 and CD133, stemness-related genes OCT4 and SOX2, EMT-related genes TWIST and NCAD, along with lower expression of epithelial gene ECAD than heterogeneous tumor cell population. Studies have confirmed the role of EMT tumor metastasis and recurrence and have a strong association with CSCs function. [19,20,21,22]. OSCC is initiated from epithelium-based stem cells that have acquired genetic alterations, such as activation and amplification of oncogenic genes or mutation in tumor-suppression genes [23,24]. Epithelial-to-mesenchymal transition (EMT) plays a vital role in initiating tumor metastasis. Though uncharacteristic gene expression patterns are supposedly pertinent to tumorigenesis of the oral mucosa, deregulated functions of some of the key promoters, interrelated with or moderated by those genes, show critical roles in the malignant transformation and cancer progression [25]. As reported by Mani et al., induction of EMT in human mammary epithelial cells could lead to mesenchymal morphology, expression of mesenchymal markers, and increased CD44 + /high/CD24−/low subpopulation cells with properties of stem cells [19]. Upon in-vitro cultivation of OSCC cells, we observed that the expression of stemness genes OCT4 and SOX2 increases with the in-vitro expansion of CD44+ oral tumor CSCs. The stemness properties of CSCs aid in perpetuation of their lineage, along with stress survival and chemotherapy resistance [26,27]. Thus, CSC-sorted cell population with increased stemness-related genes, and EMT-related genes could likely influence tumor progression and metastasis.

We also examined the conditioned media or secretome of the OSCC cells and performed a comparative analysis for the selected cytokines and growth factors. Sorted CD44+ OSCC cancer stem cells secrete higher levels of inflammatory cytokines and angiogenic factors than heterogeneous tumor cell population. Inflammation is associated with the pathogenesis of cancer [28,29]. It is projected that about 20% of the cancer cases are causatively concomitant with inflammation, particularly those of chronic cancer type [30]. Cells from tumors secrete factors related to inflammation with the purpose of causing alterations in the stroma and enable tumor invasion. Amplified inflammatory cytokine levels have been observed in numerous cancer types, including OSCC [31]. Angiogenesis is the process of formation of fresh blood vessels from primary vessels and it is necessary for maintaining the development and homeostasis of the tissues [32]. The development of the tumor also requires an accelerated angiogenesis in the niche. There are several reports stating the role of the tumor stem cells in the progression of angiogenesis during tumor formation [33]. Innovation and development of therapeutic molecules which would speed up or thwart the process of angiogenesis in the milieu of tumor development necessitates suitable pre-clinical screening. In a narrative review, Andres et al. have reported that the secretome of CSCs are not only responsible for the recruitment and activation of mesenchymal stem cells, cancer associated fibroblasts and immune cells to the tumour microenvironment but also regulate inflammatory response, tumour progressions, angiogenesis and metastasis in addition to their own maintenance [33].

The association between CSCs and tumor niche is still poorly understood. New approaches are necessary to evaluate CSCs and associated markers at various points of cancer development and metastasis, and to delineate their role in cancer progression. Additionally, there is an unmet need to simulate the entire microenvironment which mimics tumor development in the context of CSCs for experimental settings to better understand the correlation between premalignant lesions, CSCs and malignant transformations. A considerable understanding of the mechanisms regulating CSCs in aggressive OSCC and their radiation- and drug-resistance is pertinent for OSCC reversion. Novel treatment approaches must focus on novel diagnostic and prognostic modalities to combine conventional therapeutic molecules that specifically target CSCs in OSCC and their genetic regulators.

## 5. Conclusions

Sorted CD44+ OSCC stem cells express high levels of stem cell markers, stemness and EMT-related genes, exhibit higher proliferation rate and secrete higher levels of inflammatory cytokines and angiogenic factors than a heterogeneous tumor cell population from OSCC tumors. As a result, future studies are warranted to develop novel therapeutic strategies that would target both OSCC cells and CSCs, thereby improving prognosis.

## Figures and Tables

**Table 1:** PCR program used in this study.

Stage	Temperature (°C)	Time (min:sec)	Cycle
Initial denaturation	95 °C	10:00	1×
Denaturation	95 °C	2:00	
Annealing	58 °C	0:30	40×
Extension	72 °C	1:00	
Melt curve	95–60 °C	Increment of 00:05	1×

**Table 2:** List of primers.

Gene	Forward Primer	Reverse Primer
*ACTB*	5′-AGA GCT ACG AGC TGC CTG AC-3′	5′-AGC ACT GTG TTG GCG TAC AG-3′
*OCT4*	5′-GTG GAG GAA GCT GAC AAC AA-3′	5′-ATT CTC CAG GTT GCC TCT CA-3′
*SOX2*	5′-CCA GCA GAC TTC ACA TGT CC-3′	5′-ACA TGT GTG AGA GGG GCA GT-3′
*CD44*	5′-CCA GAA GGA ACA GTG GTT TGG C-3′	5′- ACT GTC CTC TGG GCT TGG TGT T-3′
*CD133 (PROM1)*	5′- CAC TAC CAA GGA CAA GGC GTT C-3′	5′-CAA CGC CTC TTT GGT CTC CTT G-3′
*TWIST1*	5′-GCC AGG TAC ATC GAC TTC CTC T-3′	5′-TCC ATC CTC CAG ACC GAG AAG G-3′
*CDH1*	5′-GCC TCC TGA AAA GAG AGT GGA AG-3′	5′-TGG CAG TGT CTC TCC AAA TCC G-3′
*CDH2*	5′-CCT CCA GAG TTT ACT GCC ATG AC-3′	5′-GTA GGA TCT CCG CCA CTG ATT C-3′

## Data Availability

The raw data supporting reported results are supplied as supplementary files.
